# Experimental and Theoretical Studies of Different Parameters on the Thermal Conductivity of Nanofluids

**DOI:** 10.3390/mi14050964

**Published:** 2023-04-28

**Authors:** Jun Qin, Yuequn Tao, Qiusheng Liu, Zilong Li, Zhiqiang Zhu, Naifeng He

**Affiliations:** 1 Institute of Mechanics, Chinese Academy of Sciences, Beijing 100190, China; 2 School of Engineering Sciences, University of Chinese Academy of Sciences, Beijing 100049, China

**Keywords:** nanofluid, thermal conductivity, thermal conductivity model, transient plane source method

## Abstract

This work experimentally investigated the effects of different factors, including nanoparticle size and type, volume fraction, and base fluid, on the thermal conductivity enhancement of nanofluids. The experimental results indicate that the thermal conductivity enhancement of nanofluids is proportional to the thermal conductivity of the nanoparticles, with the enhancement being more pronounced for fluids with lower thermal conductivity. Meanwhile, the thermal conductivity of nanofluids decreases with increasing particle size and increases with increasing volume fraction. In addition, elongated particles are superior to spherical ones for thermal conductivity enhancement. This paper also proposes a thermal conductivity model by introducing the effect of nanoparticle size based on the previous classical thermal conductivity model via the method of dimensional analysis. This model analyzes the magnitude of influencing factors on the thermal conductivity of nanofluid and proposes suggestions for an improvement in thermal conductivity enhancement.

## 1. Introduction

Since nanofluid was first introduced by Choi et al. [1] in 1995, it has attracted increasing attention in recent years. In heat transfer applications, including heat pipes [2], microchannels [3], etc., nanofluids have been widely employed to increase heat transfer efficiency. Nanofluid mainly consists of two components: base fluid and nanoparticles. When nanoparticles with a diameter of less than 100 nm are added to a base fluid at a certain concentration, the thermal and physical properties change in a big way, especially in terms of thermal conductivity. Nanoparticles, typically metal or metal oxide particles as well as carbon nanomaterials (such as CNTs and graphene oxide), can increase the thermal conductivity of water or ethylene glycol by 20–80%, as reviewed in [4]. In recent studies, carbon-based molten salt nanofluid has been shown to exhibit a remarkable improvement in thermal conductivity, ranging from 50% to 300% (see review [5]). However, the thermal conductivity of nanofluid depends on different parameters, including nanoparticle concentration, size, shape, and type, among others. Hence, it is essential to assess the effect of each parameter on the thermal conductivity of nanofluids.

A few experimental works on the measurements of the thermophysical properties of nanofluids have been reported, and relevant theoretical models have been developed to predict the correlations between nanofluid and base fluid in terms of various factors, such as particle volume concentration, particle size, particle type, and the properties of base fluid. In [6,7,8,9], when studying the effect of nanoparticle concentration on thermal conductivity enhancement, the authors found that the thermal conductivity enhancement increased nonlinearly with the nanoparticle concentration, while in [10,11,12,13,14], the correlation was found to be linear. In particular, Ceylan et al. [15] found that there exists a limit to nanoparticle loading for an enhancement in the thermal conductivity by suspending the Ag-Cu nanoparticles in pump oil. Beyond the maximum loading, the conductivity can be reduced to the base fluid thermal conductivity instead. For the effect of nanoparticle size, there has been a long-standing debate as to the relationship associated with thermal conductivity. In [16,17,18,19,20], it was found that thermal conductivity enhances with decreasing particle diameters. The main reason for this phenomenon is the mobility of nanoparticles, known as the Brownian velocity, that is inversely related to the particle size, which has been reported by Chon et al. [21]. However, the direct relationship between particle size and thermal conductivity enhancement has also been proven by many researchers [22,23,24,25]. Chen et al. [22] and Timofeeva et al. [26] suggested that the direct relationship is associated with higher thermal resistance at the particle–liquid interface and larger particle size. In addition to these two significant factors on thermal conductivity enhancement, other studies investigating the influence of other factors, such as particle type and base fluid properties, have not come to a uniform conclusion (see the reviews of [27,28]).

The prediction of the thermal conductivity of nanofluids is also a significant issue in the field of heat transfer. In examining the previous literature, most of the existing models are extensions of the classical models of Maxwell [29] and Hamilton et al. [30]. These classical models are applicable for statistically homogeneous and low-volume-fraction nanofluids with randomly dispersed, uniformly sized particles. However, the thermal conductivity is associated with the nanoscale mechanisms, and it is vital to consider the microconvections induced by Brownian motion in addition to particle diffusivity. In addition, for the nanoscale colloidal systems, there are electrostatic interactions between colloidal particles when using the electrolyte solution as the base fluid (such as molten salt) [31,32,33]. To improve the predictions, further research [34,35,36] identified and formulated the nanoscale mechanisms, including the effects of the nanoparticle–matrix interfacial layer, nanoparticle Brownian motion, and nanoparticle clusters/aggregates. Unfortunately, each of the various conclusions is typically applied to limited data, and there appears to be no agreed mechanism or theory to predict the improved thermal conductivity of nanofluids. Furthermore, none of the models mentioned above can compare the magnitude of the effect of different parameters on thermal conductivity.

In this paper, the thermal conductivity of water-based and ethanol-based nanofluids containing aluminum oxide, copper oxide, and monolayer graphene oxide was studied using the transient heat source method. This study aims to construct a novel model using dimensional analysis for assessing the magnitude of the effect of different elements, as well as to develop a semi-empirical formulation based on experimental data.

## 2. Experimental Method

### 2.1. Nanofluid Preparation

**Figure 1:**
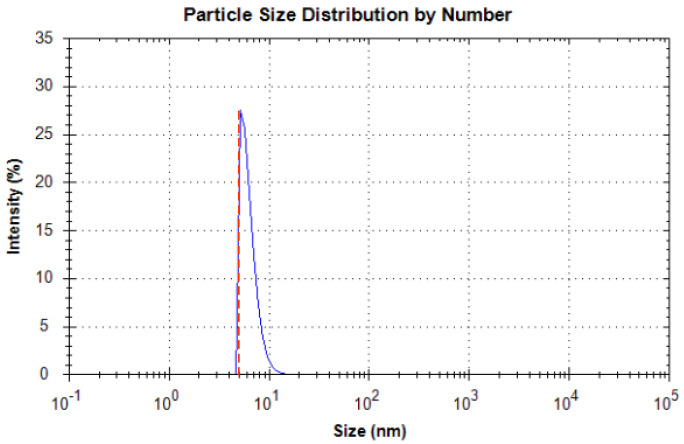
Particle size distribution of Al_2_O_3_–H_2_O with 5 nm particle size.

Nanoparticles are generally classified into three categories: metal oxide particles, metal nanoparticles, and carbon or carbide. To increase the comparability of the experiments, different commercial nanoparticles (from Xidian Chemical Technology, Tianjin, China), Al_2_O_3_ (5 nm, 50 nm, 100 nm), CuO (100 nm), and monolayer graphene oxide (thickness: 0.8 nm, 1 nm; sheet diameter: 0.5–5 μm), were chosen to prepare the nanofluids, with water and ethanol serving as the base fluids. The corresponding nanofluids were prepared using a two-step method. All chemicals were used without any additional purification. Before measuring the thermal conductivity of each sample, a Nanolink S900 nanoparticle sizer was used to check the uniformity of the particle distribution. Figure 1 shows two distinct particle size distributions of Al_2_O_3_–H_2_O nanofluids using the instrument. As seen in Figure 1, the particle size distributions of used nanofluids are all narrow and single-peaked without large particles or odd peaks. This indicates that the particles inside the nanofluids are uniformly distributed without agglomeration, which meets the experimental requirements of this study.

### 2.2. Measurement of Thermal Conductivity

**Figure 2:**
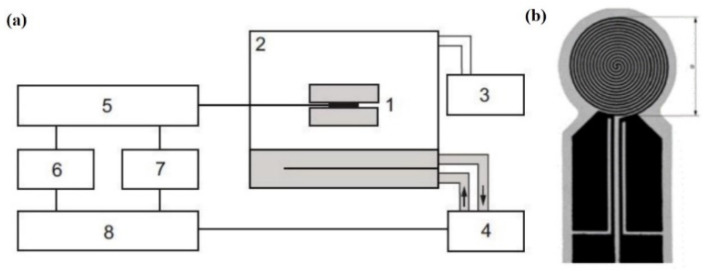
CTPS-3000 thermal constant analyzer set-up: (**a**) schematic diagram and (**b**) probe. 1—Sample and probe; 2—test chamber; 3—vacuum pump; 4—thermostat; 5—resistance measurement circuit; 6—voltmeter; 7—voltage source; 8—computer.

The thermal conductivity of the nanofluid was measured using a CTPS-3000 thermal constant analyzer. The schematic diagram of the equipment is shown in Figure 2a. It consisted of a control computer, a TPS controller, a test cell, and a probe. The probe consisted of a (10 + 2) μm thick metal foil etched into a double helix conductive structure and a very thin insulating film covering its sides (see Figure 2b). To simplify the analysis, we characterized the effect of nanoparticles on the thermal conductivity enhancement by calculating the thermal conductivity ratio $k_{n}/k_{b}$ of the nanofluid $k_{n}$ and the base fluid $k_{b}$. The measurement process of the CTPS-3000 thermal constant analyzer was as follows:

Sample pretreatment: before performing the thermal constant measurement, the probe needed to be clamped by the attached liquid cell assembly; then, the liquid to be measured was filled using a syringe through the small hole on the liquid cell to fill the cavity inside the liquid cell, and was sealed before testing.

Heat flow measurement: The heat flow measurement is the most central step of the CTPS-2500 thermal constant analyzer. The instrument uses a high-precision heat flow meter and temperature sensor to measure the heat flow of the sample. During the measurement, the sample was subjected to a thermal stimulus and the associated data were recorded using the heat flow meter and temperature sensor.

Thermal constant calculation: Based on the resulting temperature rise curve, the thermal conductivity of the sample was calculated. This calculation process was based on relevant physics models and algorithms to ensure the accuracy of the results.

Each measurement of thermal conductivity was carried out at a temperature of 20 °C and repeated three times to quantify the uncertainty. Prior to assessing the thermal conductivity of the nanofluids, the thermal conductivity of the base fluids, namely water and ethanol, was determined at 20 °C using the thermal constant analyzer. The measured thermal conductivity values were averaged, and the corresponding standard deviation was calculated (Table 1). Subsequently, the thermal conductivity of each nanofluid sample was measured at 20 °C, and the mean value and standard deviation were computed. The average value was selected as the representative thermal conductivity of the nanofluid, and the standard deviation was considered as the measurement error, which was plotted in the figures using error bars. These steps were meticulously performed for all nanofluid samples synthesized using the two-step method.

## 3. Results and Discussion

### 3.1. Effect of Different Parameters

**Figure 3:**
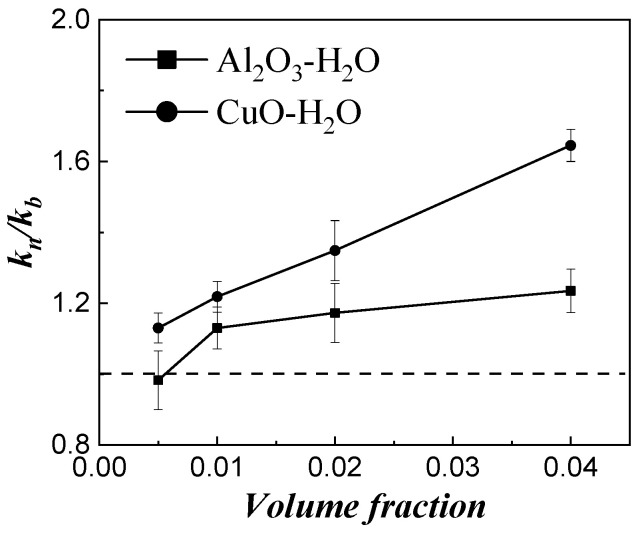
Effect of thermal conductivity of particles.

Figure 3 shows the effect of the particle material on the thermal conductivity enhancement of two oxide particles: alumina and copper oxide, with a particle size of 100 nm, both in water. All other parameters were approximately constant, isolating the material property effect. As shown, the addition of nanoparticles can effectively improve the thermal conductivity of the base fluid, and it increases with increasing volume fraction. Meanwhile, the higher conductivity particles provide a larger enhancement in the thermal conductivity, since the thermal conductivity of copper oxide is higher than aluminum oxide. It seems that the difference in the enhancing effects of the two distinct particles increases with volume fraction.

**Figure 4:**
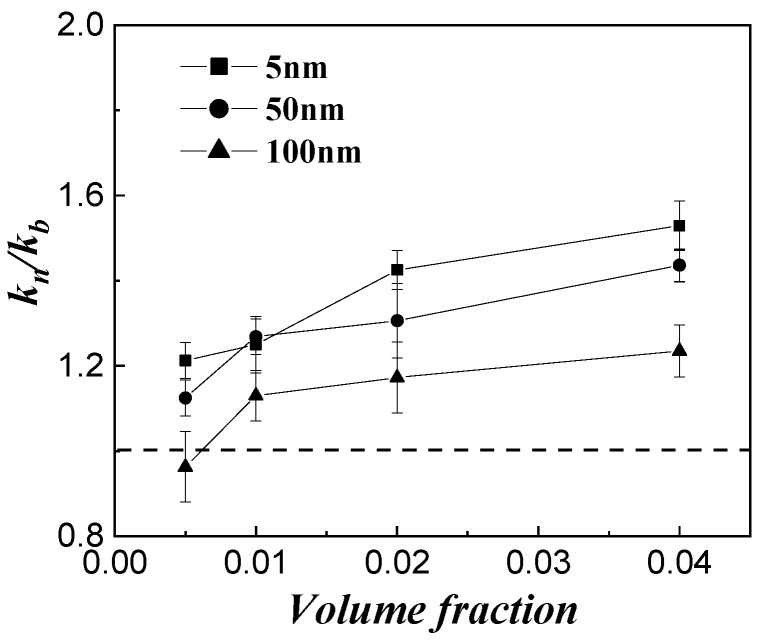
Effect of particle size of Al_2_O_3_–H_2_O nanofluids.

Figure 4 shows the influence of particle size on thermal conductivity enhancement for the Al_2_O_3_–H_2_O with different particle sizes. Here, all the particles in the nanofluid were spherical, with the diameter being the nominal diameter. As seen from Figure 4, the thermal conductivity ratio of the nanofluid gradually increases with the decrease in particle size, and the enhancement effect is more obvious at a higher volume fraction. However, at a lower volume fraction, the enhancement in thermal conductivity is not obvious. In addition, when the particle size is between 50 and 100 nm, reducing the particle size of nanoparticles can effectively improve the thermal conductivity of the base fluid. Additionally, the improvement in the thermal conductivity of the base fluid induced by the particle size change is slightly useful when the particle size is less than 50 nm.

**Figure 5:**
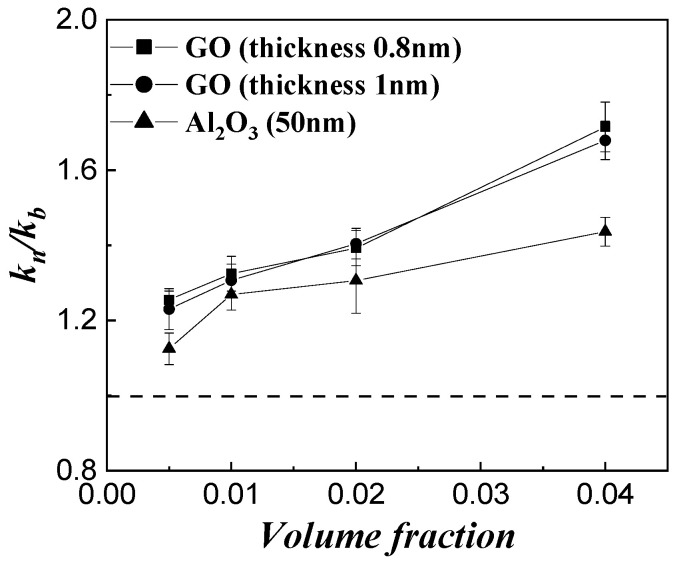
Effect of particle shape of GO and Al_2_O_3_ in water.

Figure 5 shows the thermal conductivity ratios of three different shapes of particles in water. Nanographene oxide has a monolayer mesh structure and has two shape parameters, thickness, and sheet diameters. Here, the sheet diameter of the GO particle is 0.5–5 μm. The mesh structures show a higher thermal conductivity enhancement than the spheres, and this is thought to be due to the elongated particles forming a mesh that conducts heat through the fluid. However, the trend is not clear when changing the thickness of the mesh structure.

**Figure 6:**
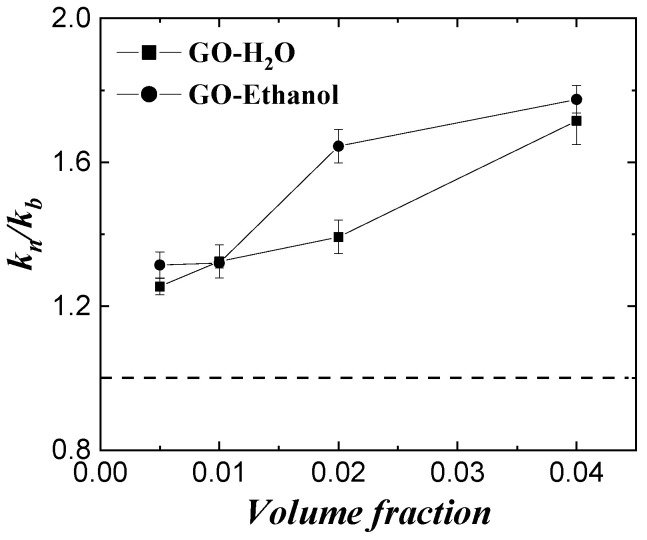
Effect of base fluid material of GO in fluids.

Figure 6 shows the effect of base fluid itself on the thermal conductivity enhancement of the monolayer graphene oxide (GO) nanofluid for two liquids: water and ethanol. From the figure, the enhancement effect of graphene nanoparticles on the thermal conductivity of ethanol is significantly higher than that of water; the addition of nanoparticles can more effectively improve the thermal conductivity of the base liquid with lower thermal conductivity, and it increases with increasing volume fraction.

### 3.2. Theoretical Model

To compare the magnitude of different parameters influencing thermal conductivity enhancement, dimensionless analysis was utilized to develop an empirical model for the thermal conductivity of nanofluids. Considering the effects of nanofluid base fluid, particle size, volume fraction, shape, particle type, etc., the empirical model of the thermal conductivity of nanofluid is given as

(1) $$k_{n}=f(d,\omega ,n,k_{b},k_{p})$$

where *d* is the particle size, *ω* is the volume fraction, *n* is the particle shape coefficient, and $k_{p}$, $k_{b}$, and $k_{n}$ are the thermal conductivity of nanoparticles, base fluid, and nanofluid, respectively. Here, we assume that the nanofluid is uniformly dispersed and ignore the effect of Brownian motion. In Formula (1), *ω* and *n* are dimensionless variables. Using Buckingham Pi theorem, the formula can be rewritten as

(2) $$k_{n}/k_{b}=\prod_{1}{}^{C_{1}}\prod_{2}{}^{C_{2}}\prod_{3}{}^{C_{3}}\prod_{4}{}^{C_{4}}$$

where *∏*_1_–*∏*_4_ are the dimensionless parameters and *C*_1_–*C*_4_ are the exponents. To obtain the dimensionless parameter of volume fraction, we simplify the classical Maxwell model [29]. The model is given by


(3) $$k_{n}/k_{b}=\frac{k_{p}+2k_{b}+2(k_{p}-k_{b})\omega }{k_{p}+2k_{b}-(k_{p}-k_{b})\omega }=\frac{1+2\frac{k_{b}}{k_{p}}+2(1-\frac{k_{b}}{k_{p}})\omega }{1+2\frac{k_{b}}{k_{p}}-(1-\frac{k_{b}}{k_{p}})\omega }$$

Since $k_{p}$ is generally much larger than $k_{b}$, $k_{b}/k_{p}$ can be approximately equal to 0. Therefore, the formula can be rewritten as

(4) $$k_{n}/k_{b}=\frac{1+2\omega }{1-\omega }$$

So, we can obtain the first dimensionless parameter *∏*_1_ of volume fraction and the second dimensionless parameter *∏*_2_ of the base material as follows

(5) $$\prod_{1}=\frac{1+2\omega }{1-\omega },\prod_{2}=\frac{k_{p}}{k_{b}}$$

Since the Maxwell model does not explain the significant enhancement in the thermal conductivity of nanofluids, Hamilton and Crosser [30] introduced the particle shape coefficient *n* based on the Maxwell model considering the effect of particle shape, and established the following thermal conductivity model for nanofluids:

(6) $$k_{n}/k_{b}=\frac{n\omega (k_{p}-k_{b})}{k_{p}+(n-1)k_{b}-\omega (k_{p}-k_{b})}$$

where *n* is the particle shape factor. Here, we conducted the same process for the H-C model; Formula (6) can be rewritten as

(7) $$k_{n}/k_{b}=\frac{n\omega }{1-\omega }$$

Formula (7) shows that $k_{b}/k_{p}$ is proportional to *n*, and *n* is directly *∏*_3_.

Both above models only consider the shape and volume fraction of the particles and ignore factors such as agglomerates, particle size, etc. Therefore, Yu et al. [37] developed a new thermal conductivity model based on the Maxwell model taking into account the particle boundary adsorption layer effect. The model is given by

(8) $$k_{n}/k_{b}=\frac{k_{p}+2k_{b}+2\omega (k_{p}-k_{b}){\left(1+2L/d\right)}^{3}}{k_{p}+2k_{b}-\omega (k_{p}-k_{b}){\left(1+2L/d\right)}^{3}}$$

where *L* is the thickness of the particle adsorption layer, usually about 1–3 nm, and *d* is the particle diameter. Conducting the same process for Formula (8), the corresponding relationship is reduced to

(9) $$k_{n}/k_{b}=\frac{1+2\omega {\left(1+2L/d\right)}^{3}}{1-\omega {\left(1+2L/d\right)}^{3}}$$

Then, the dimensionless parameter of particle size is given by

(10) $$\prod_{4}=1+2L/d$$

If one substitutes *∏*_1_–*∏*_4_ into Formula (2), it becomes

(11) $$k_{n}/k_{b}=C_{0}{\left(\frac{1+2\omega }{1-\omega }\right)}^{C_{1}}{\left(\frac{k_{p}}{k_{b}}\right)}^{C_{2}}n^{C_{3}}{\left(1+2\frac{L}{d}\right)}^{C_{4}}$$

By introducing five empirical constant values, we ultimately incorporated the volume fraction of particles, particle size, shape, type, and base fluid type into the thermal conductivity of our model.

### 3.3. Discussion

#### 3.3.1. Derivation of Exponents *C*_1_*–C*_4_

To obtain the values of exponents *C*_1_*–C*_4_, we employed multiple linear regression based on experimental data. Multiple linear regression is a statistical technique used to analyze the relationship between a dependent variable and two or more independent variables. This method is an extension of simple linear regression, which involves only one independent variables. In multiple linear regression, a linear equation is employed to model the relationship between the dependent variable and the independent variables. The equation is represented as follows:

(12) $$Y=\beta_{0}+\beta_{1}X_{1}+\beta_{2}X_{2}+\beta_{3}X_{3}+\beta_{4}X_{4}$$

where *Y* is the dependent variable, *X*_1_, *X*_2_, *X*_3_, and *X*_4_ are the independent variables, *β*_0_ is the intercept, and *β*_1_, *β*_2_, *β*_3_, and *β*_4_ are the coefficients. Here, we took the logarithm of Formula (11) to obtain the linear form:

(13) $$\ln(\frac{k_{n}}{k_{b}})=\ln C_{0}+C_{1}\ln(\frac{1+2\omega }{1-\omega })+C_{2}\ln(\frac{k_{p}}{k_{b}})+C_{3}\ln n+C_{4}\ln\left(1+2\frac{L}{d}\right)$$

Therefore, we utilized multiple linear regression by comparing it with Formula (12), where

(14) $$Y=\ln(\frac{k_{n}}{k_{b}}),X_{1}=\ln(\frac{1+2\omega }{1-\omega }),X_{2}=\ln(\frac{k_{p}}{k_{b}}),X_{3}=\ln n,X_{4}=\ln\left(1+2\frac{L}{d}\right)$$

Here, the coefficients *C*_1_, *C*_2_, *C*_3_, and *C*_4_ represent the change in the dependent variable for a one-unit change in each independent variable, while holding all other independent variables constant. These coefficients were estimated using the method of least squares, which minimizes the sum of the squared differences between the observed values of the dependent variable and the predicted values from the linear equation. To derive the expression for the nanofluid thermal conductivity ratio, a wide range of experimental data was employed, encompassing various volume fractions (0.5%, 1%, 2%, and 4%), nanoparticle sizes (5 nm, 50 nm, and 100 nm), and shape factors (*n* = 6, 9), as delineated in Table 2.

In this way, the coefficients *C*_0_, *C*_1_, *C*_2_, *C*_3_, and *C*_4_ were determined, leading to the empirical expression of the thermal conductivity model, as depicted in Formula (15). By examining the values of *C*_1_*–C*_4_ in Formula (15), we can evaluate the impact of diverse parameters on thermal conductivity.

(15) $$k_{n}/k_{b}=1.052{\left(\frac{1+2\omega }{1-\omega }\right)}^{0.1121}{\left(\frac{k_{p}}{k_{b}}\right)}^{0.0628}n^{0.0331}{\left(1+2\frac{L}{d}\right)}^{0.058}$$

#### 3.3.2. Comparison with the Previous Model

Prior research efforts related to the thermal conductivity model can be broadly classified into two principal categories: The first is the classical model, which is essentially a continuation of the Maxwell model, as discussed in Section 3.2. The second category is the dynamical model, which considers the impact of Brownian-motion-induced micromixing. Koo and Kleinstreuer [38] proposed a model that decomposes the nanofluid thermal conductivity into two distinct components, as outlined below:

(16) $$k_{n}=k_{s}+k_{B}$$

Here, *k_s_* represents the static thermal conductivity, which is predominantly derived from the classical model, while the latter *k_B_* corresponds to the enhanced thermal conductivity resulting from the convective heat transfer arising from the Brownian motion of particles and the accompanying fluid motion in the surrounding environment. Recent research endeavors have predominantly focused on *k_B_*, and as such, we present a summary of the frequently reported models in the academic literature. These models are enumerated in Table 3.

Based on the analysis presented in Table 3, it is evident that most of the existing models are primarily focused on evaluating the thermal conductivity values of nanofluids. However, these models lack the ability to compare the impact of each parameter. Although some models incorporate Brownian effects by introducing specific parameters, such as random motion velocity, accurately determining the value of such parameters continues to be a challenge, thus rendering these models less user-friendly. While we recognize the potential benefit of introducing the Brownian effect in our model, we have not yet established a satisfactory scaling approach for this purpose. Consequently, addressing this issue will be a valuable direction for future research.

## 4. Conclusions

In summary, we have systematically investigated the effect of nanoparticles on thermal conductivity enhancement. Multiple parameters such as particle volume fraction, material, diameter, shape, and base fluid properties were individually considered. Combining the experimental results and the theoretical analysis, a semi-analytical model of the thermal conductivity of nanofluids was established, which can estimate the magnitude of factors on the thermal conductivity of nanofluids. The model shows that the volume fraction has the strongest effect, the particle size and thermal conductivity of nanoparticles have a greater effect on the thermal conductivity of nanofluids, and the shape factor has the smallest effect. For enhancing the thermal conductivity of nanofluid, nanoparticles with higher thermal conductivity and smaller particle sizes should be selected, and the volume fraction of nanoparticles should be enhanced.

The findings of this research will provide a scientific foundation for better applications of nanofluids in related fields, especially in heat and mass transfer enhancement in microchannels. It is anticipated that the study will have a positive impact and serve as a source of inspiration for future studies.

## Figures and Tables

**Table 1:** Thermal conductivity of water and ethanol at 20 °C.

Liquid	Thermal Conductivity/(W/(m-K))			Average Value	Standard Deviation
Water	0.601	0.585	0.609	0.598	0.012
Ethanol	0.174	0.182	0.180	0.179	0.004

**Table 2:** Proposed experimental data for the coefficients *C*_1_*–C*_4_.

Particle Type	Volume Fraction	Particle Size	Shape Factor	Base Fluid	*k_n_/k_b_*
Al_2_O_3_	0.005	5 nm	6	water	1.21
Al_2_O_3_	0.01	5 nm	6	water	1.25
Al_2_O_3_	0.02	5 nm	6	water	1.42
Al_2_O_3_	0.04	5 nm	6	water	1.53
Al_2_O_3_	0.01	50 nm	6	water	1.27
Al_2_O_3_	0.01	100 nm	6	water	1.13
CuO	0.02	100 nm	6	water	1.35
GO	0.01	/	9	water	1.32

**Table 3:** Dynamic models for the thermal conductivity of nanofluids.

Models	Expression	Remarks
Jang and Choi [39]	$k_{n}=k_{b}\left(1-\omega \right)+k_{p}\omega +3C\frac{d_{bf}}{d}k_{b}RePr$	$d_{bf}$ is the base fluid molecule diameter, *Re* is the Reynold number of particles
Koo [40]	$k_{B}=5\times 10^{4}\omega {\left(\rho c_{p}\right)}_{b}\times \sqrt{\frac{K_{B}T}{\rho_{p}d}}f(T,\omega )$	*K_B_* is the Boltzmann constant, f(T,ω) is a function of temperature and volume fraction
Feng and Kleinstreuer [41]	$k_{B}=49500\cdot \frac{K_{B}\tau_{p}}{2m_{p}}C_{c}{\left(\rho c_{p}\right)}_{b}\omega^{2}\left(TInT-T\right)\times$ $\exp\left(-\zeta \varphi \tau \right)\sinh\left(\sqrt{\frac{{\left(3\pi \mu_{b}d\right)}^{2}}{4m_{p}^{2}}-\frac{K_{P}}{m_{p}}}\frac{m_{p}}{3\pi \mu_{b}d}\right)/\left(\tau_{p}\sqrt{\frac{{\left(3\pi \mu_{b}d\right)}^{2}}{4m_{p}^{2}}-\frac{K_{P}}{m_{p}}}\right)$	$\tau_{p}$ is the Brownian motion time interval

## Data Availability

The data is unavailable due to the privacy or ethical restrictions.
